# Looking Left: Ecologically Based Biosecurity to Prevent Pandemics

**DOI:** 10.1089/hs.2023.0089

**Published:** 2024-02-19

**Authors:** Jamie K. Reaser, Rohit A. Chitale, Gary M. Tabor, Peter J. Hudson, Raina K. Plowright

**Affiliations:** Jamie K. Reaser, PhD, was a Senior Advisor, Center for Large Landscape Conservation, Bozeman, MT, and is a Senior Scientist/Project Director, Smithsonian Conservation Biology Institute, Front Royal, VA.; Rohit A. Chitale, PhD, MPH, was Program Manager, Defense Advanced Research Projects Agency (DARPA), Arlington, VA, and is Senior Infectious Diseases Advisor, Council on Strategic Risks, Washington, DC.; Gary M. Tabor, MSc, VMD, is Chief Executive Officer, Center for Large Landscape Conservation, Bozeman, MT.; Peter J. Hudson, DPhil, is Willaman Professor of Biology, Department of Biology, Pennsylvania State University, State College, PA.; Raina K. Plowright, BVSc, MSc, PhD, is Rudolf J. and Katharine L. Steffen Professor of Veterinary Medicine, Department of Public and Ecosystem Health, College of Veterinary Medicine, Cornell University, Ithaca NY.

**Keywords:** Biosecurity, Ecological countermeasures, Land use-induced spillover, Pandemic prevention, Policy

## Introduction

Environmental conditions and human health are core contributors to national security.^1^ For decades, governments have been called to take a more comprehensive approach to biosecurity as a component of national security by broadening the concept of biological security to encompass ecological security.^2^ We define *biosecurity*, for the purposes of this commentary, as measures aimed at preventing the introduction and spread of biological organisms harmful to human assets, including human lives and livelihoods. In this context, we regard *harm* as processes that adversely impact the ability of ecological systems to generate ecosystem products (eg, fresh water, food, fuel sources) and services (eg, regulation of pests and disease). Highly influential institutions such as the World Health Organization acknowledge that “human health ultimately depends upon ecosystem products and services which are requisite for good human health and productive livelihoods.”^3^ Such acknowledgments recognize that the protection of human health is a paramount ecological service provided by nature.^4-6^

In the United States, policy actions have become increasingly consistent with the premise that a wide range of biologically based threats place the nation at risk. This is evidenced in recent executive orders and actions, including policies that regard health security and biological preparedness in the national security context^7^; connect human, animal, and environmental health within the US Global Health Security Strategy^8^; and frame the invasive species issue as a national security imperative.^9^ However, despite this policy framework, a review by the Council on Strategic Risks states that “global ecological disruption is arguably the 21st Century's most underappreciated security risk” and that “both climate and broader ecological security risks continue to be underrecognized as issues with present and tangible consequences for safety, security, and US strategic interests.”^10^

The COVID-19 pandemic brought biosecurity to the forefront of policymaking worldwide, drawing civil society's attention to the implications of “wildlife diseases” on human populations. As much as 75% of emerging *zoonoses*, infectious diseases arising from pathogens that spread from animals to humans, were initially detected in wildlife.^11^ While pathogens are a fundamental aspect of ecological systems, various perturbations can disrupt pathogen-wildlife interactions. Typically, land use change is the primary trigger for the chain of events by which zoonotic pathogens pass from wildlife to humans.^12,13^ This process includes (1) the presence of microbes that are pathogenic to humans circulating in free-living wildlife populations (pathogen host *infection*), (2) the infected host *shedding* viable pathogens into the environment (eg, via feces, urine, saliva), (3) human exposure to the pathogens occurring with subsequent infection (ie, *spillover*),^14^ and (4) further *spread* of the pathogen through the human population.^15^ We have previously termed this sequence of events the *infect-shed-spill-spread cascade* and the overall process *land use-induced spillover*.^16^ The spread of such pathogens from human to human may result in a small number of cases (clusters) or in regional (epidemic) or global (pandemic) outbreaks.

Human consumption of and trade in wildlife are closely tied to land use changes; effects on natural resources may force local people to find alternative ways to sustain themselves, and habitat degradation often enables greater human access to wildlife. Wildlife consumption and commerce must be addressed to reduce the risk of human exposure to zoonotic pathogens.^17^ However, the most fundamental approach to minimizing pandemic risk is to prevent the ecological conditions that initiate land use-induced spillover.^16^ This, from our perspective, is where biosecurity should be rooted—in the security of living systems (*bio* in Greek). Because harm to ecological systems is an integral aspect of our definition of biosecurity, we also view biosecurity as integral to biological conservation. This type of biosecurity could be achieved by (1) fostering *landscape immunity*, the ecological conditions that, in combination, keep pathogen populations in check and foster the immunological defenses of wildlife within a specific ecosystem; and (2) minimizing the risk of human exposure to zoonotic pathogens, known as managing the *dynamics of wildlife-human proximity*.^18-20^

Here we build on previous work (cited throughout this commentary) to make the case that *ecological countermeasures* (defined in the next section) provide a practical approach to achieving biosecurity at the landscape scale. We place ecological countermeasures squarely within the biosecurity context, with emphasis on the environment, defense, and health sectors, and highlight the importance of ecological countermeasures in building a more biologically informed approach to national security.

## Ecological Countermeasures

Ecologically based solutions are needed to counter land use-driven problems. Thus, there is an urgent need to consider how the condition of lands and waters affects global health security, and to recognize that one of our best defenses against future pandemics is ecological protection and restoration—measures that will enable ecological systems to be more resilient to acute and chronic perturbations. This perspective builds on and advances the options to respond to zoonotic outbreaks outlined by Everard et al,^21^ who made an explicit call to consider the foundational role of ecosystems and their services in zoonotic disease prevention, as well as to regard ecological restoration as a strategic response to zoonotic disease regulation.

For the purposes of this commentary, we regard *ecological protection* (sometimes referred to as *environmental protection*) as the act of preventing harm to ecosystem structures and functions, including ecological services. Where the protection of human health is a valued ecological service, protecting landscape immunity may be an explicit goal of ecological protection. *Ecological restoration,* in contrast, is intended to halt ecological harm and institute processes that reverse damage to an area for the purposes of recovering ecological structures and functions. For example, in 2021, we proposed the recovery of landscape immunity as an explicit goal of ecological restoration.^20^ As a related concept, *ecological resilience* can be regarded as the capacity of an ecosystem to resist and recover quickly from harm, thereby maintaining its structures and functions. Resilient ecosystems are regarded as “healthy” ecosystems.^4^ In 2022, we proposed that landscape immunity conveys resilience in ecosystems where zoonotic disease outbreaks are a potential perturbation.^19^

*Ecological countermeasures* are highly targeted, landscape-based interventions aimed at arresting one or more of the components of land use-induced spillover. Fundamentally, ecological countermeasures are an aspect of ecological restoration intended to achieve resilience to anthropogenic disturbances at the ecosystem scale.^19^ Ecological countermeasures are therefore biosecurity measures that warrant at least as much research and development investment as other technical approaches to zoonotic disease risk reduction (eg, vaccines, vector biocontrols).^22^ Without question, spillover prevention—that is, keeping zoonotic pathogens from being transmitted between species—is the most cost-efficient approach to future biosecurity policy development.^23^

Any act of ecological restoration taken to prevent zoonotic spillover by fostering landscape immunity can be considered an ecological countermeasure.^19^ At the local scale, relatively straightforward activities such as installing nesting boxes to help recover populations of vector-eating birds, or controlling invasive plants that provide ideal microclimates for pathogen vectors, can be conceived of as ecological countermeasures. The need and challenge going forward is to implement ecological countermeasures at ecosystem scale. Here, we briefly summarize 2 large-scale zoonosis-prevention projects, one aquatic and the other terrestrial, that we have conceptualized within an ecological countermeasures framework.

## Ecological Countermeasures to Prevent Zoonoses

### Schistosomiasis Prevention

Schistosomiasis is an infestation of parasitic flatworms (*Schistosoma* spp) via aquatic snail hosts (eg, invasive *Biomphalaria straminea*) that causes life-threatening health conditions (eg, anemia, liver failure, bladder cancer, and lasting cognitive impairment) in more than 250 million people in Africa, Asia, and South America, with nearly 800 million more at risk. In Africa's Senegal River Basin, reintroduction of river prawns indigenous to the west coast of Africa (*Macrobrachium vollenhovenii*), where dam construction had blocked their annual migration, could offer a sustainable, low-cost form of snail control; when this approach was used in synergy with existing drug distribution campaigns (ie, medical countermeasures), the prawns were able to reduce or locally eliminate the parasite.^24,25^ Reestablishing trophic structure by restoring river prawns throughout these river ecosystems could serve as a novel ecological countermeasure.

### Hendra Virus Prevention

Hendra virus is a fruit bat-borne virus associated with high fatality in horses and humans (with horses acting as a bridging host) in Eastern Australia. The virus was first isolated in 1994, from an outbreak involving 21 horses and 2 humans in the Brisbane suburb of Hendra, Australia.^26^ Historically, flying foxes (fruit bats; *Pteropus* spp) were nomadic across vast expanses of their native habitats, moving thousands of kilometers in accordance with tree phenology, often in response to major climatic events. However, winter food shortages due to loss of forests that provide nectar in winter, interacting with moderate to severe climate events, caused these bats to take up residence in human-dominated landscapes where they can access reliable but poor-quality foods, especially from trees planted for decoration, shade, or fruit production.^27^ The agricultural landscapes the fruit bats now inhabit include horse pastures, and thus create the potential for horses to be exposed to bat excrement near the fruiting trees where the bats feed. Bats from roosts in such pastures are responsible for almost all Hendra virus spillover events in Australia.^26,27^ However, when remnant winter-flowering forests produce flushes of nectar, the bats leave their agricultural roosting sites en masse, flying long distances to feed in what remains of their traditional forested landscapes. Every time these increasingly rare pulses of winter nectar occur, spillovers are prevented. Thus, reestablishing winter-flowering eucalyptus tree species could enable bats to return to a context that conveys landscape immunity.^27^ This enhanced winter habitat could change the dynamics of human-bat proximity by reducing bat contact with horses, as well as improve the bats' nutritional status, thereby improving bat immune capacity and decreasing viral shedding of zoonotic viruses (eg, via urination).^27^ Implementation of this ecological countermeasure is in progress.

## Ecological Countermeasure Concepts in Interrelated Sectors

### Countermeasures in Defense

From an environmental perspective, *countermeasures* typically refer to site remediation and restoration activities undertaken to address contaminants.^28^ However, the ecological countermeasures concept and term itself arose out of a project supported by the US Defense Advanced Research Projects Agency (DARPA), which focused on stopping zoonotic spillover by characterizing the dynamics of henipavirus spillover from flying foxes to humans in Asia, Africa, and Australia. DARPA's support for the project reflected an understanding by the US Department of Defense (DOD) that environmental and human health are irrevocably linked. From a defense perspective, preventing zoonotic diseases reduces the need for military intervention by, for example, preventing forced human migration and conflict over limited resources.^29^ In addition, military personnel are especially at risk of disease when they occupy degraded environments, and they may place other people at risk by spreading pathogens or their vectors when relocating. DARPA's investment in the henipavirus spillover project also reflects DOD's charge to take the conservation actions necessary to “sustain the long-term ecological integrity of the resource base and the ecosystem services it provides.”^30^

Multiple DOD agencies, in addition to DARPA, execute innovative conservation- and human health-relevant research and development via projects facilitated by several grantmaking programs. In 2022, DOD issued a sustainability plan that recognizes that “to successfully execute this mission, the Military Departments must have access to the energy, land, air, water and other natural resources necessary to develop, train, and operate—today, and in the future. The Department recognizes the reality of an emerging climate crisis that is impacting our installations, equipment, and forces.”^31^ The concept and practice of ecological countermeasures is thus consistent with the military mission-space, at least within the United States.

The term *ecological countermeasure* is also in alignment with military linguistic frameworks: DOD defines *countermeasure* to mean “that form of military science that, by the employment of devices and/or techniques, has as its object the impairment of the operational effectiveness of enemy activity.”^32^ This definition recognizes countermeasures not as single interventions, but as a body of science that applies a diverse array of technical tools and approaches to problem resolution—that is, to impede a potentially harmful agent from becoming harmful. It, therefore, stands to reason that ecological countermeasures, like any other form of countermeasure, should be applied to national security. This can be exemplified by the notion of landscape immunity, an ecological condition that minimizes the risk of zoonotic spillover—with spillover being the “enemy activity” to be prevented.

### Countermeasures in Health

In the context of a broad national security agenda that links environmental condition to human wellbeing, it is also important to note that the term *countermeasure* is already well established in the health sector and is thus a key concept to be integrated into multiple US and global strategic documents, including the US Global Health Security Agenda^33^ and the US National Health Security Strategy and Implementation Plan.^34^ Medical countermeasures include lifesaving medicines and medical supplies used to diagnose, prevent, or treat conditions associated with chemical, biological, radiological, or nuclear threats, emerging infectious diseases, or natural disasters.^35^ The US Centers for Disease Control and Prevention uses the term *medical countermeasure* (eg, vaccines, antiviral drugs) within their public health and emergency preparedness framework^36^ to guide their capacity to provide medical interventions when a public health incident occurs. This work is complemented by the US Food and Drug Administration Medical Countermeasures Initiative, which coordinates medical countermeasure development, preparedness, and response.^37^ The World Health Organization has also adopted the medical understanding of countermeasures; for example, “sufficiency of countermeasures” is one of the primary criteria used to determine if a pathogen is of substantial health risk. Indeed, many zoonotic diseases are considered among the greatest public health threats due to their epidemic potential and countermeasure insufficiencies.^38^

## Advancing Ecological Countermeasure Science

In 2021, we established a 5-point action plan to address the needs and opportunities to further elucidate land use-induced spillover and establish ecological countermeasures as a component of ecological restoration.^20^ Given that ecological resilience conveys biosecurity, this proposal is readily applicable to advancing ecological countermeasure science through a biosecurity lens. Here, we succinctly summarize this high-level proposal for advancing ecological countermeasure science:

1. One Health and Planetary Health frameworks are ideal contexts for collaborative, interdisciplinary ecological countermeasure concept development, case study cataloging, and research collaboration.^39,40^ Although every ecological countermeasure project needs to be fit-to-context in terms of approaches and timelines, a next step in this field of work is to develop risk analysis frameworks that can be used to assess the costs and benefits of interventions, identify opportunities to amplify returns on investment where multiple goals can be achieved simultaneously (eg, conserving biodiversity and protecting public health), and plan for the mitigation of unintended adverse consequences.
2. Despite increased investments for zoonotic pathogen discovery in understudied species and regions, there remains a need to educate policymakers, funding agencies, and early-career scientists on these information gaps—with the goal to inspire development of the resources and sizable body of researchers needed to identify and employ ecological countermeasures.
3. Untangling the causal relationships between land use change and zoonotic spillover will require the coupling of field-based empirical studies that identify the parsimonious links with large-scale experiments and dynamic mechanistic models. Thus, there is a need for granting agencies to prioritize the funding that makes these complex investigations possible over adequate timescales.
4. Readily accessible zoonotic pathogen/host datasets are fundamental to ecological countermeasure development. National governments and multilateral frameworks should therefore set targets that prioritize zoonotic pathogen surveillance and monitoring, especially considering the pending shifts in species' geography due to globalization and climate change.
5. Public health interventions often neglect context-specific social, cultural, and historical factors and inadequately engage the people they are designed to benefit. To be effective, ecological countermeasures development and implementation must consider inputs from social science disciplines, as well as the expertise gained by Indigenous and local peoples.

## Advancing Ecological Countermeasure Policy

In principle, biosecurity draws from multiple fields, sectors, and agencies, particularly those with missions related to the management of harmful, or potentially harmful, biological organisms (eg, invasive species). One Biosecurity has been proposed as a framework to improve scientific and policy integration across the full spectrum of biosecurity concerns driven by the biological invasion of organisms, including zoonotic pathogens and their hosts.^41^ We have already addressed countermeasures in environmental, defense, and health sector contexts as historically relevant for the points made and reflecting the current highly fragmented policy paradigm within the United States. In this section, we align with a more comprehensive, integrated approach to biosecurity policy that would include ecological countermeasures as one “tool” in a comprehensive “toolkit.”

Biosecurity policy development in the United States has lagged behind that of other countries, most notably Australia and New Zealand.^42,43^ New Zealand was the earliest adopter of a comprehensive approach to biosecurity; its 1993 Biosecurity Act^44^ provides a rigorous framework for preventing harmful organisms from entering the country. New Zealand's strong commitment to enforcing this legislation, and the nearly 3 decades that its civil society has had to adopt prevention measures as societal norms, undoubtedly played a significant role in the country's ability to rapidly and robustly respond to COVID-19 in the first year of the pandemic, in a manner approachable by few other countries.^45^

We cannot identify a national security issue in the United States in which there is a greater disparity between the scale of effect and the scale of response than biological invasions, of which nonnative zoonotic pathogens are a major component. At the level of the US Executive Office of the President, the call for greater comprehensive attention to biosecurity has existed since the Carter-era executive order on exotic organisms,^46^ and has since been repeated in 2 executive orders on invasive species, in which the National Invasive Species Council (NISC) was established and then expanded to institute a cost-efficient, cooperative leadership.^9,47^ NISC now includes the secretaries of DOD and the US Department of Health and Human Services, as well as the senior-most political appointees of 10 other agencies and 4 components of the Executive Office of the President (ie, The White House).

NISC management plans and other guidance documents^48^ place a strong emphasis on prevention measures, but almost exclusively from the border control perspective. Recently, a more holistic vision was reflected in a special issue of *Biological Invasions* that responds to NISC's 2016-2018 management plan.^49^ For example, Burgos-Rodríguez and Burgiel^50^ reviewed the patchwork of US authorities that unevenly address various aspects of the federal government's legislative capacity to rapidly detect and respond to infectious pathogens, nonnative pathogen hosts, and all other invasive species, with a view toward addressing framework gaps. After considering the recommendations arising out of all of the articles in the special issue, Reaser^51^ drafted a blueprint that outlines policies, goals, and actions to be taken by relevant Executive Branch agencies and components of the Executive Office of the President to institute a national biosecurity program that engages agencies with missions ranging from border control and defense to human health and conservation. (The role of land management agencies in biosecurity should be readily apparent.) We also believe that ecological countermeasures must be a core component of this framework; reestablishing landscape immunity following environmental perturbations and managing wildlife-human dynamics of proximity will minimize the risk of biological invasion by all nonnative taxa. This is critically important given the role that a wide range of invasive plants, insects, arthropods, and vertebrates play in amplifying zoonotic disease risks.^52^

From its outset in 2021, the Biden administration has voiced an intent to address pandemic risks and interrelated national security agendas, including by prioritizing an update to the National Biodefense Strategy.^53^ The president stated that

*it is essential that we refresh and reinvigorate our national science and technology strategy to set us on a strong course for the next 75 years, so that our children and grandchildren may inhabit a healthier, safer, more just, peaceful, and prosperous world. This effort will require us to bring together our brightest minds across academia, medicine, industry, and government—breaking down the barriers that too often limit our vision and our progress, and prioritizing the needs, interests, fears, and aspirations of the American people*.^54^

While biosecurity is now unusually high on the US national security agenda, there is yet good reason to draw attention to what remains lacking; an ecological approach to addressing zoonotic disease is still largely absent from these policy constructs. For example, the *National Strategy for the COVID-19 Response and Pandemic Preparedness*^55^ is focused on medical countermeasures and safeguarding the economy. Although it does include a goal to restore US leadership and build better preparedness for future threats, measures to safeguard ecological resilience (eg, via landscape immunity) are noticeably absent from a list of measures to “build better biopreparedness and expand resilience for biological threats”—a list that includes monitoring current and emerging biological threats, securing funding to improve biopreparedness, establishing the Center for Forecasting and Outbreak Analytics at the US Centers for Disease Control and Prevention, and developing a sustainable US infrastructure for biological and pandemic events. Neither the executive order to mobilize the US response to COVID-19 and provide leadership on global health security^56^ nor the executive order to protect public health and the environment, as well as restore science to tackle the climate crisis,^57^ point to the critical role of ecological systems in addressing these issues or direct agencies to take supportive, ecologically based actions. Even within an executive order focused on the climate crises,^58^ the only explicit directive focused on ecological resilience is an echoing of the widely popularized “30 by 30” goal, which calls for saving at least 30% of US land and water by 2030.^59^ Across the full suite of pandemic-related executive actions, we also note the tendency to place agencies such as the Department of the Interior, which has a mission to conserve and manage the nation's natural resources, at the leadership periphery.

An understanding of land use-induced spillover and the deployment of ecological countermeasures is crucial to future biosecurity policy development. Protection of ecological systems goes far beyond values in aesthetics, outdoor recreation, and long-term access to the natural resources that support the human enterprise. The protection of human health is an ecological service, and COVID-19 has aptly demonstrated that site-specific measures to maintain and restore landscape immunity should be regarded as biosecurity measures of national priority and global-scale importance. Our assertion here is consistent with the “8 pillars of action” that the Council on Strategic Risks proposes to address the security implications of ecological disruption—particularly Pillar 2 to “promote methods that protect and expand critical systems and services,” Pillar 5 to “reduce pandemic risk at point of origin,” Pillar 6 to “amplify ecological and national security issues in the US government,” and Pillar 7 to “initiate an ecological security research agenda.”^10^ It is our hope that future actions spurred by the council's findings and recommendations will explicitly incorporate ecological countermeasures in policy and practice.

This commentary focuses on US biosecurity because that is the context in which we have explicit expertise. However, the overarching message that biosecurity should include landscape-based interventions—ecological countermeasures—to prevent the emergence and movement of zoonotic pathogens clearly has global relevance. In the modern age, the implications of landscape decisions made at the local level can transcend ecological and jurisdictional boundaries via trade and transportation pathways. The biosecurity principles and practices presented in this commentary need to be taken up by, among others, the International Zoonoses Community of Experts, convened by the Group of 7 ministers responsible for climate and the environment to facilitate scientific and technological collaboration,^60^ as well as the international partnership initiatives prompted by the COVID-19 pandemic, such as PREZODE (Preventing Zoonotic Disease Emergence)^61^ and the Preventing Pandemics at the Source coalition.^62^ Readers interested in advancing the science of ecological countermeasures to better inform policy can find the basis for a One Health research agenda in articles that informed this commentary.^16,19^

## Conclusion

The large-scale protection and restoration of natural systems affords adaptation and resilience capacity—which is what literally enables Earth to sustain humans and all other species. A resilient biosphere is generative in the face of tremendous pressures, including crises that facilitate human conflicts. All the options and opportunities that humans will have available to them as the world changes in unpredictable and unprecedented ways are ecologically based. In the context of zoonotic disease outbreaks, ecological countermeasures should be considered fundamental components of the national security agenda. Performance metrics for ecological countermeasures, guided by the outputs of the action plan developed by us in 2021,^20^ could serve as land management standards worldwide.

We therefore emphasize the importance of using the concept and term *ecological countermeasures* to help normalize zoonotic disease risk management as a core component of national security and to emphasize the importance of prioritizing ecologically oriented concerns and solutions among competing policy issues. Furthermore, it is our hope that frameworks and terms that explicitly make the connection between national security and its ecological foundations will help government administrations, in the United States and elsewhere, increase their support for senior-level representatives from the life sciences within national security bodies and as external advisors. Until this becomes standard practice, governments will not be able to effectively address the threats to ecological systems that subsequently drive the need for humanitarian and military interventions. Ecological resilience really is our best defense.
